# Stage and Gene Specific Signatures Defined by Histones H3K4me2 and H3K27me3 Accompany Mammalian Retina Maturation In Vivo

**DOI:** 10.1371/journal.pone.0046867

**Published:** 2012-10-09

**Authors:** Evgenya Y. Popova, Xuming Xu, Andrew T. DeWan, Anna C. Salzberg, Arthur Berg, Josephine Hoh, Samuel S. Zhang, Colin J. Barnstable

**Affiliations:** 1 Department of Neural and Behavioral Sciences, Penn State University College of Medicine, Hershey, Pennsylvania, United States of America; 2 Penn State Hershey Eye Center, Hershey, Pennsylvania, United States of America; 3 Department of Neurobiology, Yale University School of Medicine, New Haven, Connecticut, United States of America; 4 Department of Epidemiology and Public Health, Yale University, New Haven, Connecticut, United States of America; 5 Bioinformatics Core, Penn State University College of Medicine, Hershey, Pennsylvania, United States of America; 6 Department of Public Health Sciences, Penn State University College of Medicine, Hershey, Pennsylvania, United States of America; 7 Department of Ophthalmology and Visual Science, Yale University School of Medicine, New Haven, Connecticut, United States of America; Leibniz Institute for Age Research - Fritz Lipmann Institute (FLI), Germany

## Abstract

The epigenetic contribution to neurogenesis is largely unknown. There is, however, growing evidence that posttranslational modification of histones is a dynamic process that shows many correlations with gene expression. Here we have followed the genome-wide distribution of two important histone H3 modifications, H3K4me2 and H3K27me3 during late mouse retina development. The retina provides an ideal model for these studies because of its well-characterized structure and development and also the extensive studies of the retinal transcriptome and its development. We found that a group of genes expressed only in mature rod photoreceptors have a unique signature consisting of de-novo accumulation of H3K4me2, both at the transcription start site (TSS) and over the whole gene, that correlates with the increase in transcription, but no accumulation of H3K27me3 at any stage. By *in silico* analysis of this unique signature we have identified a larger group of genes that may be selectively expressed in mature rod photoreceptors. We also found that the distribution of H3K4me2 and H3K27me3 on the genes widely expressed is not always associated with their transcriptional levels. Different histone signatures for retinal genes with the same gene expression pattern suggest the diversities of epigenetic regulation. Genes without H3K4me2 and H3K27me3 accumulation at any stage represent a large group of transcripts never expressed in retina. The epigenetic signatures defined by H3K4me2 and H3K27me3 can distinguish cell-type specific genes from widespread transcripts and may be reflective of cell specificity during retina maturation. In addition to the developmental patterns seen in wild type retina, the dramatic changes of histone modification in the retinas of mutant animals lacking rod photoreceptors provide a tool to study the epigenetic changes in other cell types and thus describe a broad range of epigenetic events in a solid tissue *in vivo*.

## Introduction

Histone modifications are sensitive indicators, and possibly predictors, of gene expression, but the complexity of the epigenetic code is far from understood [1], [2], [3], [4]. Methylations of various lysine residues of histone H3 have been correlated with gene activation and with gene repression [5], [6], [7], [8], [9], [10], but the interplay between the modifications and gene expression during development is not clear, particularly in solid tissues [11]. In human T cells all three states of methylation at lysine 4 (K4) were positively correlated with transcription levels and served as marks of active genes [5]. Trimethylation of lysine 27 (H3K27me3) on the other hand was a mark of inactive genes [5]. In ES cells many genes have promoters with both K4 methylation and K27 methylation [12], [13]. Since fewer such bivalent promoters are found in differentiated cells it is thought that such bivalent domains lose one or other mark during development as selected subsets of genes become transcriptionally active or repressed. During neural development genes associated with neurogenesis and function in differentiated neurons show high levels of H3K27me3 in progenitors but this mark is lost in a lineage-specific manner upon gene activation [14].

The retina begins as an early compartment of the forebrain and has frequently served as a model of CNS development [15], [16], [17], [18], [19]. Retinal cell types are formed in a characteristic sequence from E12 to PN5 with ganglion cells, amacrine cells and horizontal cells among the early formed types and rod photoreceptors and bipolar cells formed predominantly during the later postnatal period. Several studies have described the retinal transcriptome, and the full developmental expression of over 7,600 UniGenes has been described [20], [21]. A variety of methods have been employed to refine these analyses and provide the beginnings of cell type transcriptomes [22], [23], [24], [25], [26], [27], [28], [29].

In the present study we have used ChIP-seq to map the distribution of two important histone H3 modifications, dimethylation of lysine 4 (H3K4me2) and trimethylation of lysine 27 (H3K27me3), over the whole genome at multiple time points during late mouse retina development. We have merged these data with previous expression profiles and shown that there is not a simple correlation between gene expression and epigenetic marks. A major conclusion of this study is that the epigenetic signatures defined by H3K4me2 and H3K27me3 during development can distinguish cell-type specific genes from genes with different cell type and cell lineage patterns of expression.

## Results

### Dynamic Profiles of H3K4me2 and H3K27me3 During Retina Organogenesis

**Figure 1:**
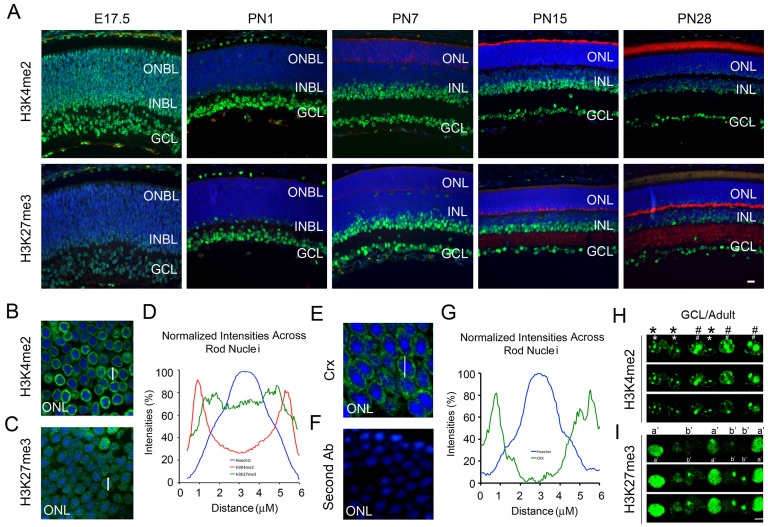
Changes in histone modifications during retina maturation. (**A**) Immunofluorescence microscopic images of sagittal sections of developmental mouse retina tissue array stained with anti-H3K4me2 (**A**, green, upper panels) and anti-Rhodopsin (**A**, red, upper panels), anti-H3K27me3 (**A**, green, lower panels) and anti-SVP38 (**A**, red, lower panels), and counterstained with Hoechst in cell nuclei (**A,** blue). ONBL, outer neuroblast layer; INBL, inner neuroblast layer; GCL, ganglion cell layer; ONL, outer nuclear layer; INL, inner nuclear layer. Scale bar represents 25 µm. (**B**) H3K4me2 labeling with high magnification for ONL from adult retina (Scale bar = 6 µm). (**C**) H3K27me3 labeling with high magnification for ONL from adult retina (Scale bar = 6 µm). (**D**) Averaged and normalized intensity profiles for fluorescence of each specific antibody or Hoechst staining across the nuclear centers (e.g. white bars in **B** and **C**) of rod photoreceptor nuclei (n = 5). (**E**) Adult retina outer nuclear layer labeled with an antibody recognizing Crx. (**F**) Control labeling of adult ONL showing lack of staining with secondary antibody alone. (**G**) Averaged and normalized intensity profile for Crx labeling (green) and Hoechst (blue) across rod nuclear centers (bar in **E**). (**H, I**) Confocal images with high magnification for RGCL from adult retina (Scale bar = 15 µm). Cells marked * or # show distinct cellular distribution with H3K4me2 antibody (**H**, green) and a′ or b′ show distinct cellular distribution with H3K27me3 antibody (**I**, green).

To determine the gross developmental changes in level and distribution of H3K4me2 and H3K27me3 we labeled a tissue array containing mouse eyes from ages E17.5 (embryonic day) to PN28 (postnatal day). At low power H3K4me2 (Figure 1A, upper panels) was initially distributed throughout the retina and from PN4 onwards showed stronger staining in cells of the ganglion cell layer (GCL) and the inner part of the inner nuclear layer (INL) with multiple intense foci in each nucleus. The mature outer nuclear layer (ONL), consisting almost entirely of rod phtoreceptor nuclei, showed much weaker staining, however a higher magnification view detected a thin ring of fluorescence just beneath the nuclear membrane (Figure 1B). H3K27me3 staining appeared later than H3K4me2 and had a more uniform distribution across the nuclei of the inner retina (Figure 1A, lower panels). In the mature ONL H3K27me3 was also only detectable at high magnification where it appeared as a diffuse ring with many speckles (Figure 1C). Measurement of the fluorescence intensities across the equators of adult rod photoreceptor nuclei confirmed that H3K4me2 was almost exclusively in the thin rim of euchromatin on the nuclear periphery (Figure 1D, red). H3K27me3 fluorescence was less intense and its distribution was broader across the nucleus with increased staining in the apocentromeric zone (Figure 1D, green) as previously described [30], [31], [32]. When adult retinal sections were labeled with an antibody against Crx, a major rod photoreceptor transcription factor, the pattern of labeling was identical to that seen for H3K4me2 (Figure 1E&G). Negative controls showed no labeling (Figure 1F). In the retinal ganglion cell layer H3K4me2 (Figure 1H) and H3K27me3 (Figure 1I) each gave two distinct patterns of nuclear labeling that may reflect the two major cell types in this layer, ganglion cells and displaced amacrine cells.These patterns are very different from those seen in the mature rods and may reflect the different organization of heterochromatin and euchromatin in these cells. These histological results suggest dynamic patterns of histone modifications that correlate with retinal cell types during differentiation.

**Figure 2:**
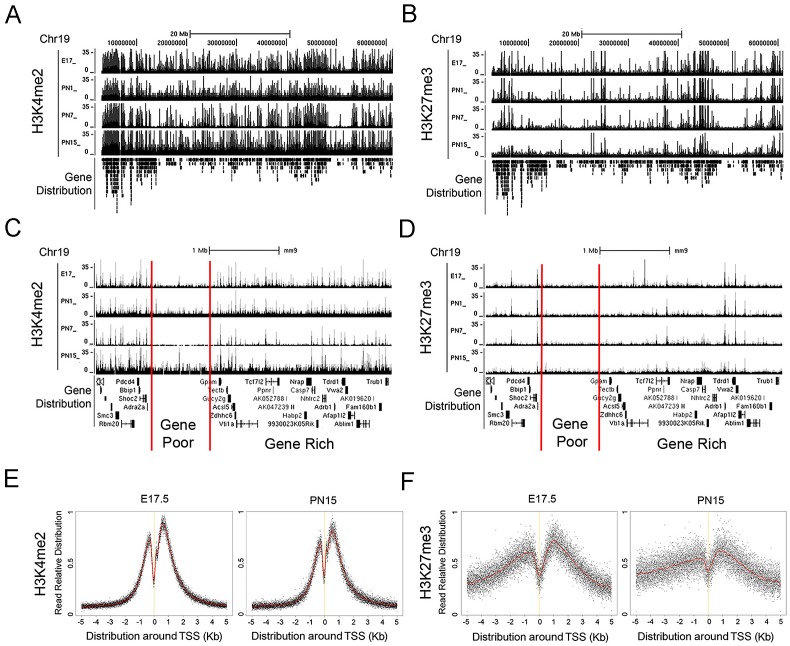
Histone modifications show an association with genes and their TSS. (**A–B**) Histone H3K4me2 (**A**) and H3K27me3 (**B**) modification patterns at part of chromosome 19. Peaks of normalized sequenced tags from ChIP-Seq analysis of mouse retina at 4 developmental stages (E17, PN1, PN7, and PN15) were mapped to mouse genome (see Text S1). Scale bar represents 20 Mb. (**C–D**) High-resolution view of the part of chromosome 19 patterns shown in (**A**) and (**B**) with gene poor or rich regions. Scale bar represents 1 Mb. Y-axis in **A–D** represents the number of reads in a 100 bp interval. (**E–F**) Normalized tag counts of histone modifications by H3K4me2 (**E**) and H3K27me3 (**F**) for all NCBI RefSeq genes around TSS (+/−5 Kb) at E17.5 and PN15. Red lines are average tag counts around TSS.Y-axis in **E** and **F** represents the number of reads in a 100 bp interval.

To explore the locations of the histone modifications in more detail we created a comprehensive genomic map using standard ChIP-Seq [33], [34]. Across all chromosomes both H3K4me2 (Figure 2A) and H3K27me3 (Figure 2B) modifications were depleted in the centromeric and telomeric regions at all developmental stages and elsewhere were localized preferentially in the gene-rich areas as exemplified by a region of chromosome 19 (Figure 2C, D). The accumulation of H3K4me2 reads in the genome were primarily localized in the area around the transcription start site (TSS), showing two sharp peaks (Figure 2E) of enrichment approximately +/−1 Kb surrounding the TSS as defined for the 25,158 genes from the NCBI RefSeq database [35], including splice variants and alternative TSS. The peaks of H3K27me3 were less pronounced and were even less distinct at PN15 than at E17.5 (Figure 2F). The occupancy by H3K4me2 or H3K27me3 in the area +/−2.5 Kb of each TSS was determined for each developmental stage and is listed in Table S1. A small set of genes from this large collection was used for confirmation by ChIP-qPCR analysis (Figure S1) and the results perfectly matched those of the ChIP-Seq experiments.

### One Fifth of All Genes have No H3K4me2 and H3K27me3 Accumulation in Retina

**Figure 3:**
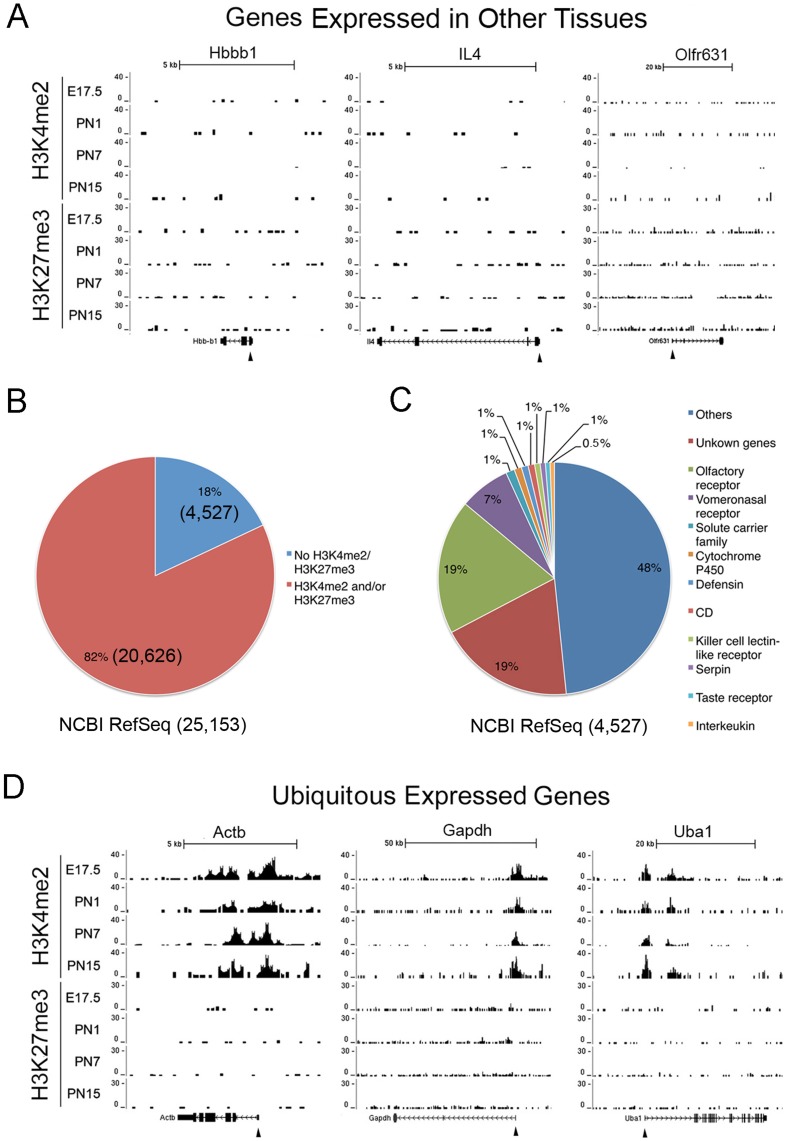
Epigenetic signatures correlate with gene expression patterns. (**A**) H3K4me2 and H3K27 accumulation in examples of genes specifically expressed in other non-retinal tissues. (**B**) Percentage of the genes lacking both H3K4me2 and H3K27me3 accumulation (18%, 4,527 genes) at TSS regions (+/−2.5 Kb) through all developmental stages from all RefSeq genes (totally 20,626 genes) tested by an analysis of Euclidean raw distances (see Text S1). (**C**) Categorizes genes (4.527 genes in **c**) lacking both H3K4me2 and H3K27me3 accumulation at TSS by their biological function and tissue specificity. Top 10 categories of genes are listed and “others” (48%) are genes with GO annotations and not listed in the pie chart; “unknown” (19%) are genes without GO annotations and not listed in the pie chart (see Table S2). (**D**) H3K4me2 and H3K27 accumulation in examples of genes ubiquitously expressed in most tissues. Y-axis represents the number of reads in a 100 bp interval.

We next investigated specific classes of genes to determine whether there was any correlation of their expression pattern with epigenetic signature. First we studied genes expressed in other tissues or cells, but not retina, such as erythrocyte specific hemoglobin (Hbb-b1), the cytokine gene IL4, and the olfactory receptor gene Olfr631, and they showed no H3K4me2 or H3K27me3 occupancy at any developmental stage (Figure 3A). To explore this in more detail we took all genes listed in RefSeq (Table S1) and used Euclidean raw distances to identify genes lacking both H3K4me2 and H3K27me3 around the TSS. The analysis identified 4,527 genes (18%) of 25,158 total RefSeq genes that had no H3K4me2 or H3K27me3 accumulation above the background value determined from anti-GFP controls (Figure 3B). Within this cluster were sets of genes encoding olfactory receptors, vomeronasal receptors, and taste receptors as well as interleukins, defensins, and lectin receptors (Figure 3C). These, and most of the other genes in the overall cluster, are tissue or cell specific in tissues other than retina (Table S2). For a more detailed analysis, we examined all 1,003 known olfactory receptor genes [36]. None of them showed any H3K4me2 accumulation and 94% had no H3K27me3 accumulation at any of the four developmental points (Table S3). These findings support our conclusion that both H3K4me2 and H3K27me3 are absent around the TSS of genes that are not expressed during maturation of retina and suggest that H3K27me3 modification is not an obligatory inhibitory mechanism for genes in terminally differentiated cells.

For comparison we next examined a group of genes expressed throughout maturation in the retina as well as by many other cell types. Examples of these widely expressed genes, beta actin (Actb), glyceraldehyde-phosphate dehydrogenase (Gapdh), and ubiquitin-like modifier activating enzyme 1 (Uba1), are illustrated (Figure 3D) and show consistent occupancy by H3K4me2 but not H3K27me3 throughout retinal development.

### Different Histone Signatures for Retinal Genes with the Same Gene Expression Pattern

**Figure 4:**
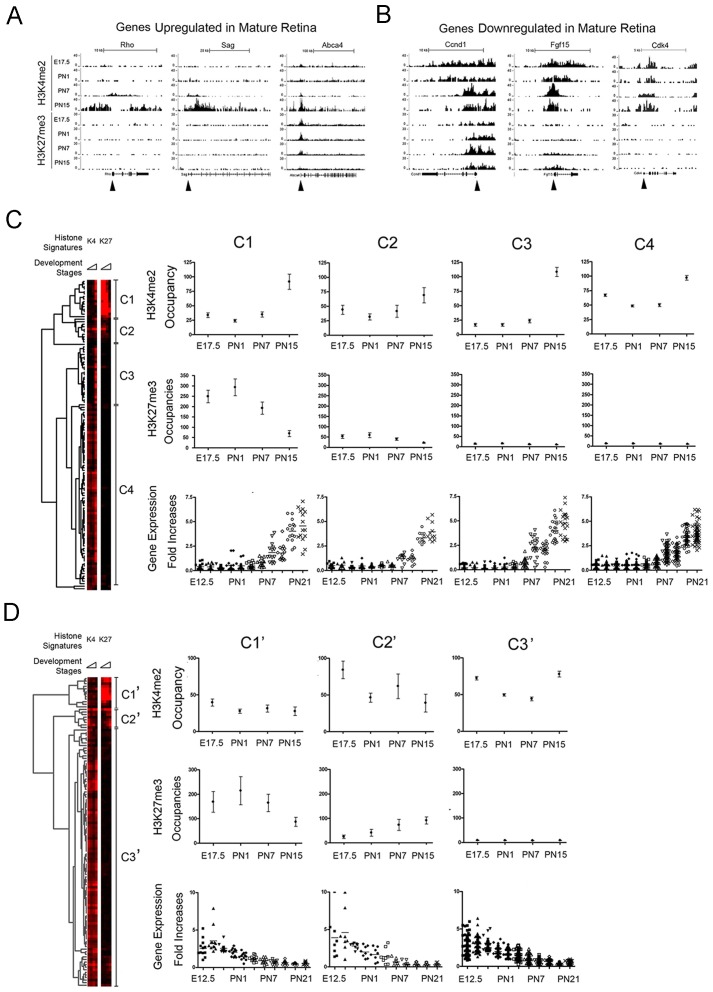
Genes with the same expression patterns show different histone signatures in retina. (**A**) H3K4me2 and H3K27 accumulation in examples of genes up-regulated during retina maturation. (**B**) H3K4me2 and H3K27 accumulation in examples of genes down-regulated during retina maturation. Closed arrowheads show TSS of each gene (see Text S1 and Tables S4 &S5). (**C**) Cluster analysis of H3K4me2 and H3K27me3 occupancy around TSS (+/−2.5 Kb) at all developmental stages for the genes upregulated in mature retina. Tree-view shows 4 clusters (C1–C4) with distinct epigenetic patterns for H3K4me2 (upper panel) and H3K27me3 (middle panel) but with same expression patterns (lower panel). (**D**) Same analysis as in (**C**) for the genes downregulated in mature retina. Cluster analysis of H3K4me2 and Tree-view shows 3 clusters (C1′–C3′) with distinct epigenetic patterns for H3K4me2 (upper panel) and H3K27me3 (middle panel) but with same expression patterns (lower panel). For **A** and **B** y-axis is the number of reads in a 100 bp interval. For **C** and **D**, (upper panels) y-axis is the normalized occupancy, or the number of reads for a given histone modification in an interval +/−2.5 kb around the TSS of given gene, normalized for the total number of mapped reads in given experiment.

In a previous study [21] of the mouse retina transcriptome we identified a pool of 123 genes whose expression increased in parallel with the maturation of rod photoreceptors (Table S4) and a pool of 119 genes whose expression was down regulated between E16.5 and PN15 (Table S5). Examples of histone modification patterns for these two different groups of genes are shown at Figure 4A, B. When we compared H3K4me2 occupancy in the TSS of the upregulated genes in wild type retina to *rd1/rd1* retinas that had lost rod photoreceptors [37], the *rd1/rd1* samples showed significantly lower occupancy (>2 fold, *P* = 2.31e-30), confirming that these genes are all rod dependent. By hierarchical cluster analysis [20], [21], [38] of the occupancy of H3K4me2 and H3K27me3 around the TSS during development, these upregulated genes fell into 4 distinct clusters (Figure 4C) and the downregulated genes fell into three clusters, although over 80% fell into a single cluster (Figure 4D).

To understand the relationship between gene expression and histone modifications in vivo, we examined the clusters of upregulated genes represented in Figure 4A, C. For genes in clusters C1 and C2 H3K4me2 accumulation matches activation of these genes during retina development and H3K27me3 accumulation is inversely proportional to gene expression. However, genes in clusters C3 and C4 have no H3K27me3 accumulation through late retinal development. H3K4me2 accumulation in these two clusters showed two different patterns, a late de novo accumulation in cluster C3 from PN7 to PN15, and a consistent but lower level of accumulation at all stages in C4. We conclude that while genes may have indistinguishable expression profiles through late retina development, these profiles may be accompanied by very different patterns of H3K4me2 and H3K27me3 modifications.

A similar disconnect between histone modification patterns and expression was observed for the genes down regulated during retina development as shown in Figure 4B, D. For example,the small cluster C1′ showed a low constant level of H3K4me2 and a declining but still substantial accumulation of H3K27me3 whereas cluster C2′ showed a decreasing trend of H3K4me2 and increasing trend of H3K27me3 across the ages tested. The large group of genes in C3′ showed a maintained accumulation of H3K4me2 without any significant accumulation of H3K27me3 at any stage. Functional analysis of genes in cluster C3′ showed an enrichment of genes involved in cell proliferation (Table S6). This strongly suggests that repression of genes in the retina does not always require a decrease in H3K4me2 and increase in H3K27me3.

### A Unique Signature of H3K4me2 and H3K27me3 Marks Rod Photoreceptor-specific Genes

**Figure 5:**
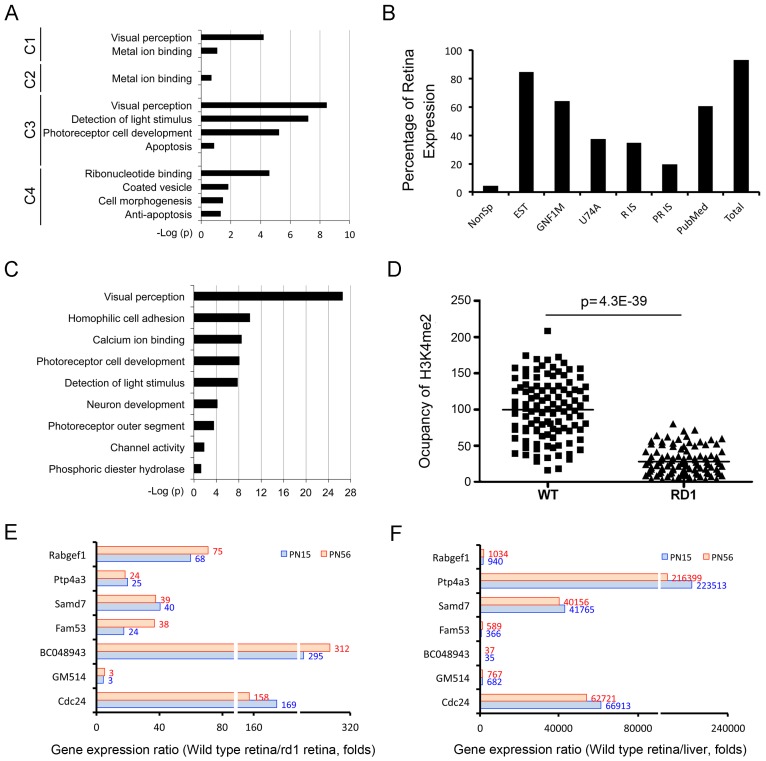
A unique signature of H3K4me2 and H3K27me3 marks rod photoreceptor-specific genes. (**A**) Functional enrichments for the gene clusters in Figure 4C identified using a web-based DAVID classification tool and GO categories (see Table S6). Cluster C3 shows very significant Benjamin P values for functions associated with photoreceptors. (**B**) The percentage of genes that have retina expression as defined in listed gene expression databases in a group of genes identified from all RefSeq genes with the same epigenetic signature as C3 in Figure 4C (see Table S8). (**C**) Functional enrichments for the genes from (**B**) using the same DAVID classification tool (see Table S9). This group of genes has a very significant Benjamin P values for functions associated with photoreceptors. (**D**) A significant reduction of H3K4me2 occupancy around TSS (+/−2.5 Kb) of the gene group from (**B**) in retinas from *rd1/rd1* mice compared to the occupancy in wild type retinas.y-axis is normalized occupancy or number of reads for a given histone modification in an interval +/−2.5 kb around the TSS of a given gene, normalized for total number of mapped reads in given experiment.t-Test for two-sample assuming equal variances was applied. P = 4.3E-39. (**E)** Ratio of gene expression levels in wild type and rd1 retinas for 7 genes identified in the unbiased cluster analysis (Table S8) at PN15 and PN56. (**F)** Ratio of gene expression levels in retina and liver for 7 genes identified in the unbiased cluster analysis (Table S8) at PN15 and PN56.

To further explore the differences among the clusters shown in Figure 4C, the functional classes of genes in each cluster were analyzed (DAVID v6.7 [39]) using Gene Ontology (GO) categories. C2 and C4 contained genes that are expressed in many cell types in retina and other tissues (Figure 5A). C1 and C3 contained genes functionally grouped into visual function or development (Figure 5A). Cluster C1 contained genes associated with neurons or the immune system, except for three genes expressed in rods as well as other cells, and shows an increase in H3K4me2 and a decrease in H3K27me3 between E17 and PN15, exemplified by Abca4 in Figure 4A. C3, on the other hand, contained a group of highly specific photoreceptor genes (Table S7) and had a unique combined signature of de-novo increase of H3K4me2 beginning at PN7 and a lack of H3K27me3 occupancy at any stage (Figure 4A, C). As shown in Figure 5A, genes with this unique epigenetic signature are significantly enriched, 44–117 fold, in the functions of visual perception, light detection, and rod development.

To test whether this signature might have predictive power we extended the cluster analysis to all 25,158 genes from RefSeq database, and identified a group of 113 genes, including almost all of C3, that showed the same late de novo increase of H3K4me2 and a complete lack of H3K27me3. Five of these genes were excluded from further analysis because they showed large amounts of non-specific signal as defined with the control anti-GFP antibody. 95% of the remaining genes showed expression in eye by a variety of methods (Figure 5B, Table S8). These included the well-characterized photoreceptor-specific genes such as Rho, Crx, Nrl, Nr2e3, as well as the mouse orthologues of recently identified photoreceptor disease related genes such as BC027072 [40], [41], Gm11744 [42]. The analysis also included genes such as Ppef2, Lrit1 and Lrit2 that have been implicated in phototransduction but whose exact function has yet to be determined. Similarly, DAVID analysis (Figure 5C) identified this group as genes with significant enrichment in photoreceptor functions (Table S9) including visual perception, light detection, rod development. The occupancy by H3K4me3 at the gene TSS in this group is significantly reduced in *rd1/rd1* compared to wild type retina (Figure 5D), further supporting that the genes are photoreceptor specific. To provide a direct test of the expression of some of these genes we selected 7 and measured expression in wild type and *rd1/rd1* retinas by quantitative PCR (qPCR). As shown in Figure 5E, the ratios of wild type/rd1 expression were all substantial. We also measured the ratios of expression in wild type retina and liver and again found strong positive ratios (Figure 5F). Both seta of data add further confirmation of the rod specificity of these genes and support our conclusion that a specific epigenetic signature is associated with cell type-specific genes.

### Do Other Retinal Cell Type-specific Genes have Distinct Epigenetic Signatures?

**Figure 6:**
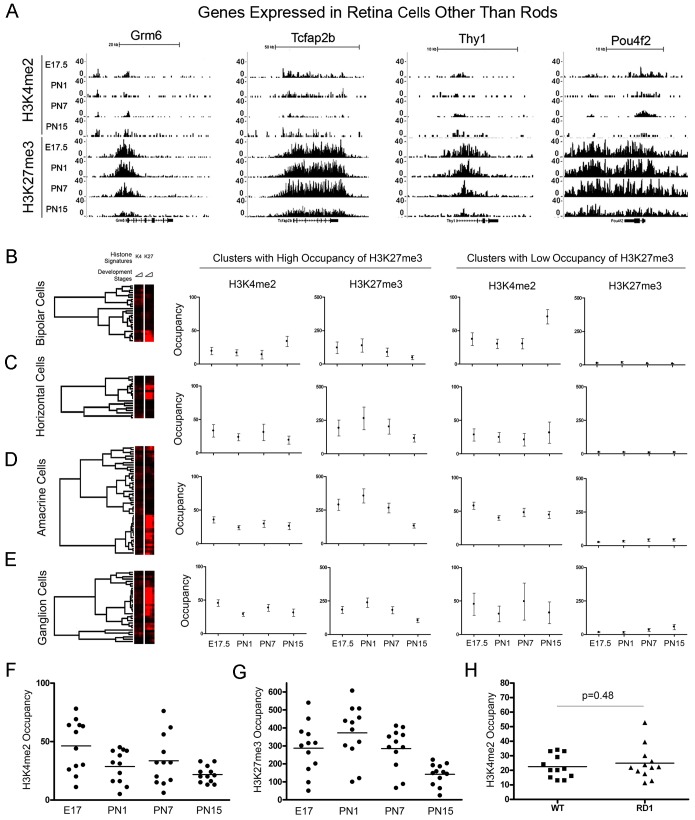
Distinct epigenetic signature for genes specifically expressed in retina but not in photoreceptors. (**A**) H3K4me2 and H3K27 accumulation in the examples of genes specifically expressed in bipolar cells - Grm6, amacrine cells – Tcfap2b; ganglion cells – Thy1 and Pou4f2. See Table S10 for details. (**B–E**) Cluster analysis of H3K4me2 and H3K27 occupancy at all developmental stages around TSS (+/−2.5 kb) for the genes highly expressed (combine Table S11) in bipolar (**B**), horizontal (**C**), amacrine (**D**), and ganglion cells (**E**). Tree-views show 2 clusters with either high (left panels) or low (right panels) H3K27me3 occupancy around TSS for each group. Dot-plot charts of H3K4me2 (**F**) and H3K27me3 (**G**) accumulation for the genes highly expressed in retina cell types other than rod photoreceptors (Table S10). (**H**) Equal H3K4me2 occupancy around TSS (+/−2.5 Kb) for the genes expressed in retina cell types other than rod photoreceptors ()in wild type and mutant*rd1/rd1* mice (P = 0.48). For (**A**) y-axis is number of reads in a 100 bp interval. For (**B** to **H**) y-axis is normalized occupancy or number of reads for given histone modification in interval +/−2.5 Kb around TSS of given gene, normalized for total number of mapped reads in given experiment.

The mature retina is dominated by rod photoreceptors and these cells provide the bulk of the epigenetic signals detected in our ChIP-seq experiments. A variety of studies, however, have positively identified a group of genes highly expressed in specific retinal cell types, but not rod photoreceptors (Table S10). Examples from this group representing bipolar-, amacrine- and ganglion cell-specific genes are shown in Figure 6A. These genes show a substantial H3K27me3 accumulation from the earliest stages of development and a small but distinct accumulation of H3K4me2 around the TSS, as summarized in Figure 6 F and G. The occupancy of H3K4me2 within these cell groups was unchanged following the loss of rods in *rd1/rd1* retinas (Fig. 6H, *P*>0.05).We then combined these with a set of genes reported [29] as highly expressed in specific retinal cell types (Table S11) and used a hierarchical cluster analysis on the whole group. For each cell type we found that two clusters could be defined according to high or low accumulation of H3K27me3 (Figure 6B–E). Within the H3K27me3 high occupancy group, these showed small but distinct accumulations of H3K4me2. Interestingly, all the genes that are positively identified as specific to a single retinal cell type fall into the cluster of high H3K27me3 occupancy (Table S11). We conclude that genes highly specific for individual non-rod photoreceptor retinal cell types have specific signatures characterized by high levels of H3K27me3 around the TSS and lower, but distinct, accumulations of H3K4me2.

### Gene-wide Coverage by H3K4me2 and H3K27me3 is a Landscape for Gene Activity

**Figure 7:**
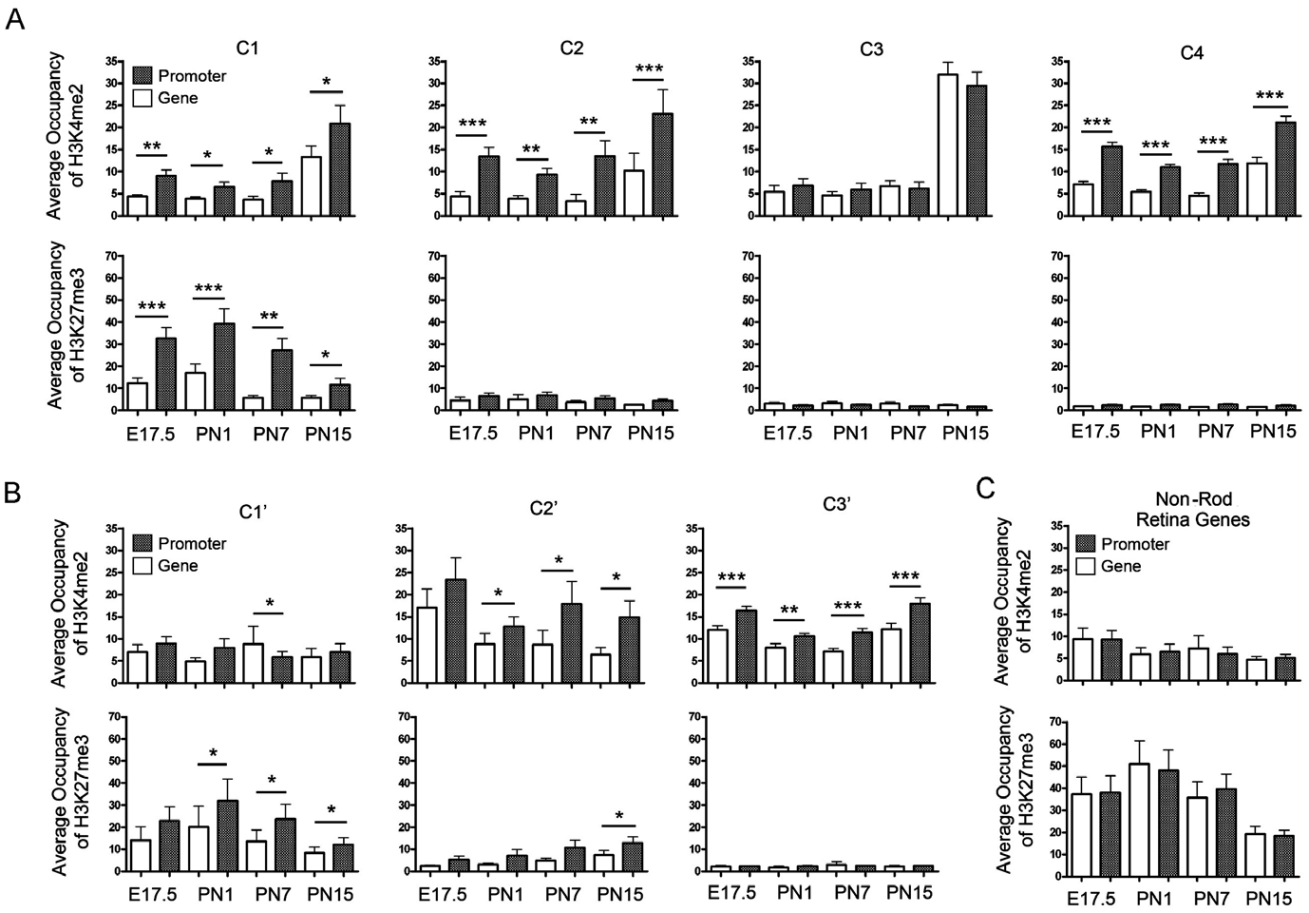
Gene-wide coverage by H3K4me2 and H3K27me3 as a landscape for maintenance of gene activities. (**A**) Comparison of average normalized occupancy per 1 Kb of H3K4me2 (upper panels) and H3K27me3 (lower panels) between promoter region (from −2.5 Kb to TSS) and whole gene body (from TSS to TES) for the clusters from Fig. 4c, C1 to C4 (see Table S12). (**B**) Same comparison as in (**A**) for the clusters from Fig. 4d, C1′ to C3′ (see Table S13). (**C**) Same comparison as in (**A**) for genes specific expression in retina but not photoreceptors (see Table S14). Y-axis is average occupancies for genes in given cluster, where occupancy is (number of reads in an interval×5,000,000 reads×1 kb)/(total number of reads in experiment × length of genome interval in kb).t-Test for two-sample assuming equal variances was done to compare occupancy on promoter and gene body for each stage and * marks those with statistically significant differences: (*) 0.05>*P*>0.01, (**) 0.01>*P*>0.001, (***) *P*<0.001 (see details in Text S1).

All the analysis above focused on the transcription start site. For a number of genes, however, we noticed developmental changes in the accumulations of H3K4me2 and H3K27me3 over the whole gene. We tested whether the occupancies of H3K4me2 or H3K27me3 normalized over the full length of the genes were similar to the occupancies in promoter regions (0 to −2.5 kb) for the clusters shown in Figure 4C (also Table S12). Of all the clusters, only the rod-specific genes showed a good correlation of H3K4me2 accumulation at the promoter and the gene body (Figure 7A, C3). All the clusters of downregulated genes (Table S13) showed significantly lower accumulation of H3K4me2 and H3K27me3 over the gene as compared with the promoter (Figure 7B). The genes expressed in specific retina cells other than rods (TableS14) display a strong correlation of H3K27me3 accumulation at promoter and gene body through development (Figure 7C, *P*<0.05 or greater). In contrast to TSS-centered profiles, occupancy over the gene body by H3K4me2 or H3K27me3 as well as their promoter might serve as specific epigenetic signatures associated with tissue or cell specific genes, suggesting a role in maintenance of the active or repressive state at the end of differentiation.

## Discussion

**Figure 8:**
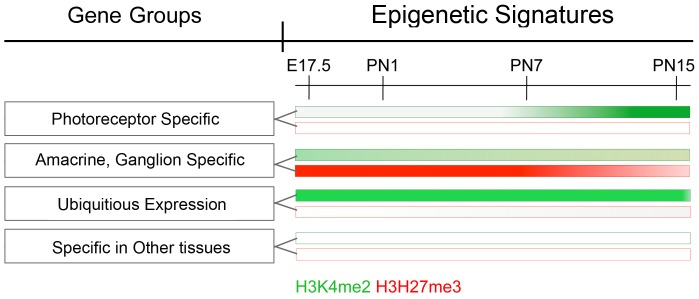
Schematic representation of H3K4me2 and H3K27me3 epigenetic signatures for different gene groups during retina maturation. Four gene groups (left panels), representing photoreceptor specific expression, amacrine/ganglion cell specific expression, ubiquitous expression, and expression specific in other tissues are shown in this scheme. Each gene group has different epigenetic signatures with changing levels of H3K4me2 (green) and/or H3K27me3 (red) accumulation on gene TSS through the retina maturation from E17.5 to PN15. The color intensities represent levels of histone accumulation.

During postnatal retinal maturation there is a dramatic change in the pattern of gene expression [21] with downregulation of genes such as those associated with proliferation and an upregulation of genes associated with the terminally differentiated cell types. Our results clearly show that each of the various categories of genes has characteristic epigenetic signatures and that these can be defined by just two histone modifications, H3K4me2 and H3K27me3. These various classes of genes and the changing pattern of histone modifications are represented diagrammatically in Figure 8.

Perhaps our most significant finding was that rod photoreceptor-specific genes had an unambiguous epigenetic signature of a de novo accumulation of H3K4me2 and no H3K27me3. We initially found this by determining the signature around the TSS of a set of genes that we had previously defined as rod-specific or rod-dependent [21]. We then identified this signature among all the genes in the NCBI RefSeq database. This unbiased analysis identified a large gene cluster that included almost all of the genes we know to be rod photoreceptor-specific. Among those genes, BC027072 is the mouse orthologue of recently described disease gene C2ORF71 associated with retinitis pigmentosa (RP) in human [40], [41]. Another gene Gm11744, also known as PRCD, has a defined mutation in human and dog RP [42].For other genes that are less well characterized, a variety of information in expression databases and our qPCR analysis both support our conclusion that this cluster represents a set of rod photoreceptor-specific transcripts. Such epigenetic signatures may be a powerful tool to identify new cell type-specific genes, particularly those that are not expressed at high levels and might be missed by most expression-based methods. Shared promoter sequences, or common transcription factors, have so far failed to identify the whole class of rod photoreceptor-specific genes. The finding of a distinct epigenetic signature for rod photoreceptor-specific genes that is not found in other genes expressed in rods suggests that there is some precise spatial or temporary regulation of the enzymes controlling histone methylation. There is growing evidence for a role of transcription factors in initiating the assembly of complexes that include enzymes capable of modifying histones, but whether these include enzymes creating the modifications examined in this study has yet to be determined. Genome wide ChIP-Seq data for two major retina transcription factors Crx [43] and Nrl [44] is available only for adult retina, so temporary patterns for binding of these factor during development and its correlation with developmental epigenetic changes is subject for future study.

A second group of genes expressed in retinal neurons, but not rods, had low but detectable accumulations of H3K4me2 and much larger accumulations of H3K27me3 (Figure 8). While it is possible that some of the H3K27me3 accumulation may represent repression of these genes in rod photoreceptors, we suspect that much of it is due to crosstalk among the cells of the inner retina because the histological results of Figure 1E showed the highest levels of labeling for H3K27me3 in the inner retina.

During retinal maturation we identified a third group of genes expressed in many cell types that had heavy accumulations of H3K4me2 but no H3K27me3. This is in contrast to the fourth class of genes, those never expressed in retina that had no accumulation of either H3K4me2 or H3K27me3 at any developmental stage. Such a finding may provide a sensitive diagnostic criterion for whether genes are ever expressed in the retina. Similarly, we would predict that rod-specific genes, such as Opsin or Sag, should have no accumulation of either H3K4me2 or H3K27me3 in other tissues. Although there are only limited data in accessible databases, this appears to be the case since in a ChIP-seq analysis of the C2Cl2 myogenic cell line neither the Opsin nor the Sag gene showed any accumulation of either H3K4me2 or H3K27me3 (LICR Histone Track, UCSC Genome Informatics). Similarly, the set of olfactory receptor genes that showed no H3K4me2 or H3K27me3 in our retinal data also had none of these marks in the C2Cl2 cell line. As further data become available it will be possible to test whether our finding can be generalized over many cell types.

Previous studies using cultured cells have suggested that an observed increase in H3K4me2 “premarks” genes for upregulation [14], [45], but in the intact solid tissue we found that the increase in H3K4me2 reached its full magnitude well after the onset of gene expression. In our previous analyses of opsin transcription we found that the increase in opsin RNA could be divided into two phases [21], [46], [47]. The early phase is due to the initial transcription of the gene in an increasing number of rod photoreceptors, and the larger later phase to increased transcription in the final complement of cells.The relatively late H3K4me2 accumulation that we observed at the opsin TSS could correspond to the second phase of upregulated expression, although our methods may not detect accumulation in small populations of rod photoreceptors at early postnatal stages.

It was recently suggested that genes specifically expressed in blood cells displayed high levels of H3K4me2 over the whole gene bodies in addition to the TSS, in sharp contrast to TSS-centered profile typical of housekeeping genes [45], [48]. Our results extend this idea and we propose that the occupancy over the gene body by H3K4me2 or H3K27me3 as well as their promoter is part of the specific epigenetic signatures of tissue or cell specific genes and may reflect possible roles in maintenance of active or repressed state in the end of differentiation.

We also did not find a good temporal relationship between H3K27me3 accumulation and induced gene expression. Although some genes did show an inverse correlation between H3K27me3 accumulation and level of gene expression, others showed falling levels of H3K27me3 during development as the levels of gene expression decreased. This suggests that H3K27me3does not serve as a universal early repressor that is removed at the onset of transcription. This differs from the finding in progenitor - neuron transitions in culture where more than half the upregulated neuron-specific genes lost H3K27me3 marks upon differentiation [14] or in myoblast-myotube transition in culture where a large group of permanently repressed genes maintained the mark throughout myogenesis [49]. Other studies have linked H3K9me2, H3K9me3 and H4K20me3 accumulation with permanent suppression of genes and so it is possible that H3K27me3 is involved in only a specific step of gene suppression rather than serving as a general marker.

In this study we have provided the first example of epigenetic modifications during late development of a solid tissue in vivo. In addition to the developmental patterns seen in wild type tissue, we can observe dramatic changes in the retinas of mutant animals. While many of these may be due to the altered cellular composition in the degenerating tissue, more careful analysis may reveal other and earlier changes indicating effects of the disease on epigenetic patterns in the affected tissue. As similar data become available for other tissues, the combination of gene expression data for specific cell types with modified histone signatures will offer unique insights into the regulation of different gene classes and may lead to a broader understanding of epigenetic codes during organogenesis.

## Materials and Methods

### Mice

All animal experiments were conducted in accordance with NIH and ARVO guidelines and were approved by the Animal Care and Use Committee of Pennsylvania State University School of Medicine (Protocol # 2006-080 and 2009-061). C57BL/6j and *rd1/rd1*-Pde6b-RD1 (*rd1/rd1*) mice [37] were purchased from the Jackson Laboratory (Bar Harbor, ME). The *rd1/rd1* mutant mice were used as rod photoreceptor subtraction control.

### Antibodies and Reagents

Chemicals were purchased from Fisher Scientific (Pittsburgh, PA), unless otherwise noted. Opsin [50] and SVP38 [51] monoclonal antibodies have been described previously. Anti-H3K4me2 (07–030) and anti-H3K27me3 (07–449) were from Upstate (Charlottesville, VA) and were used before for ChIP [13], [52] and passed validation (htt://compbio.med.harvard.edu/antibodies/) [53]. Anti-GFP antibodies were from Santa Cruz. Validated antibodies (Figure S2) were used for ChIP to prepare libraries with IlluminaChIP-Seq analysis.

### Immunohistochemistry

Methods were as previously described [54]. Antigen retrieval was achieved by boiling samples 5 min in 10 mM citrate buffer pH 6.0. Sections were labeled with primary antibodies and secondary antibodies conjugated with FITC (*Molecular Probes*) or Texas Red (*Jackson ImmunoResearch*Inc.). Digital images were recorded using Olympus fluorescence or Olympus FV1000 confocal microscopes.

### Nuclei Isolation for ChIP-Seq and ChIP-qPCR

20 mouse retinas were rapidly isolated and rinsed in PBS on ice. Cell suspensions in PBS were crosslinked with 1% formaldehyde for 15 min at room temperature, followed by quenching with 1 M glycine, incubation on ice for 5 min, and centrifugation for 7 min at 4,000 rpm at 4°C. For nuclei isolation the pellet was resuspended in 1 ml RSB buffer (10 mMNaCl, 3 mM MgCl_2_, 19 mM Hepes, pH 7.5), with 1 mM PMSF, 10 ul protease inhibitors (PI) and 0.5% Igepal CA-630 (Sigma), incubated on ice for 20–30 min and centrifuged at 6,000 rpm for 7 min at 4°C.

For ChIP-Seq, nuclei were resuspended in 1 ml RSB (PMSF+PI) and DNA concentrations measured spectrophotometrically. Micrococcal nuclease (MN) test digestions were carried out to determine the time interval needed to produce predominantly mononucleosomes and this was used for preparative digestion. For preparative micrococcal nuclease digestion, nuclei (0.5 mg/ml DNA) was resuspended in 1 ml RSB, 0.5 mM PMSF, 1 mM CaCl_2_, 2.5 units/ml MN, incubated at 37°C for 45–60 min and terminated by 5 mM EDTA. Nuclei were centrifuged for 7 min at 7,500 rpm, pellet was resuspended in 500 ul L-CHIP buffer (1% SDS, 10 mM EDTA, 50 mM Tris-HCl pH 8.0), 1 mM PMSF and PI, sonicated twice at setting 3 for 10 sec on Sonic Dismembrator (Fisher Scientific, Model 100). The pellet of mononucleosomes was subjected to ChIP after pre-cleaning by centrifugation at 14,000 rpm for 5 min. Protein concentration was adjusted to 1 mg/ml with L-CHIP buffer. For ChIP-qPCR samples were treated similarly except that sonication to shear DNA to lengths of between 200 and 2,000 bp was used.

### Chromatin Immunoprecipitation

Chromatin was diluted 10 fold in D-CHIP buffer and 5 ug antibody was added and incubated with rotation overnight at 4°C. Simultaneously 30 ul protein A beads (Sigma) slurry were washed 2 times in washing buffer with 9∶1 of D-CHIP (dilution buffer: 0.01% SDS, 1.1% Triton X-100, 1.2 mM EDTA, 16.7 mM Tris-HCl pH 8.0, 167 mM NaCl) and L-CHIP, resuspended in the same buffer with 500 ug/ml salmon sperm DNA (Invitrogen) and 100 ug/ml BSA (Invitrogen) and incubated on rotator overnight at 4°C. Beads were washed 2 times with washing buffer, combined with the chromatin/antibody mix and rotated for 2 hours at 4°C. Beads were washed 4 times with 1 ml LS-CHIP buffer (low salt buffer: 0.1% SDS, 1% Triton X-100, 2 mM EDTA, 20 mM Tris-HCl pH 8.0, 150 mM NaCl), 1 time with 1 ml HS-CHIP buffer (high salt buffer: 0.1% SDS, 1% Triton X-100, 2 mM EDTA, 20 mM Tris-HCl pH 8.0, 500 mM NaCl) and eluted with 350 ul of E-CHIP buffer (elution buffer: 1% SDS, 0.1 M NaHCO_3_) by rotating at room temp for 10 min. Immunoprecipitate (IP) and 50 ul of input chromatin (Input) were treated with 0.5 mg/ml of Proteinase K (Roche) and RNaseA (Roche) at 37°C for 30 min and uncrossliked at 65°C overnight. DNA was extracted twice with phenol/chloroform, once with chloroform and ethanol precipitated with glycogen (Roche) and sodium acetate. DNA was dissolved in 50 ul water and subjected to qPCR or was used to prepare libraries with IlluminaChIP-Seq DNA Sample Preparation Kit (IP-102-101). Quality and quantity of DNA in ChIP-Seq libraries were validated on Agilent Technologies 2100 Bioanalyzer. Libraries were sequenced on an Illumina Cluster Station and Genome Analyzer at Genomic Resource Center of The Rockefeller University.

### qPCR

Quantitative real-time PCR was done according to Illumina Protocol Guide for qPCR Quantification (Illumina, Cat. # SY-930-1010) using primers listed in Table S15. Triplicate samples were run on an iQ5 Multicolor Real Time PCR Detection System (Bio-Rad).

### Data Preparation

We used program Bowtie (version 0.12.4) to map all sequence reads to the mouse genome NCBI37/mm9. Sequence reads mapped to multiple genomic locations were excluded from subsequent analysis. The mid point of each unique-mapped reads was calculated with the assumption that all fragments are 190 bp in size and were used for subsequent analysis. One Bedfile was created for each sample by counting number of unique-mapped reads in each 100 bp-window along all chromosomes normalized to the total unique-mapped reads in the sample. The final Bedfiles were uploaded to a website, and custom tracks were created in UCSC Genome Browser with links pointing to the real Bedfiles, that were used for specific gene mapping. All sequencing data have been deposited in Gene Expression Omnibus (GEO) data repository under accession number GSE38500.

### Tag Distribution Analysis in Defined TSS Region

To create the tag distribution around the TSS, we downloaded genomic data of RefSeq [35], [55] (http://hgdownload.cse.ucsc.edu/goldenPath/mm9/database/refGene.txt), and counted the number of unique-mapped reads located in TSS +/−5 Kb. Then the number of reads at each position was divided by the number of total reads in their regions. Smoothing function LOWESS in R package was used to calculate a smoothed density curve.

### Detection of Tag Enriched Region in Genome

We applied a Poisson distribution-based model to detect enriched regions. We tested all windows in whole chromosome with pace of 100 bp, windows with p-value below 1.0e-5 were collected as enriched, and overlapping windows were merged into single enriched region. We also tested the same enriched region on anti-GFP control samples and removed those regions from the enriched list if they were also significantly enriched in the GFP positive samples.

### Detection of Tag Enriched Location in Individual Genes

The reads from each experiment were mapped to mouse genome and analyzed with NextGENe software (version 2.10). Bed format files for specific genes were made for promoter area from +2.5 Kb to TSS or for whole body of the gene from TSS to TES and amount of reads were calculated for each genome interval at each developmental stage with NextGENe software. Normalized occupancy for each genome interval = (amount of reads×5,000,000 reads×1,000 Kb)/(whole amount of reads in experiment×length of genome interval in Kb).

### Gene Functional Analysis

The Database for Annotation, Visualization and Integrated Discovery (DAVID [39], [56]) v6.7 web-accessible programs (http://david.abcc.ncifcrf.gov) was used for GO functional analysis. Unigene ID of each gene from specific clusters is inputted into the web-based program each time to obtain gene-GO term enrichments score and p values under a default Searching Algorithm. The EASE Score and Benjamini values are used to evaluate gene functional enrichments.

### Analysis of Genes without H3K4me2 and H3K27me3 Accumulation in Retina

To have a better cutoff for the genes without H3K4me2 and H3K27me3 accumulation, a cluster of the normalized H3K4me2 and H3K27me3 dataset of the E17, PN1, PN7, and PN15 time-points were created using MatLab version R2011b based on Euclidean row distance. The average of the GFP controls (value of 9.12) was used as a background and normalized as an Euclidean distance of 8 dimensions (value of 25.80) to determine the cutoff of the Euclidean row distances indicating genes with no H3K4me2 or H3K27me3 hits.

### Statistical Analysis

Analysis was done with Excel Data Analysis (Microsoft Excel 2007) and GraphPadPrizm 4 software. Cluster analysis [38] was done with Gene Cluster 3.0 software with hierarchical clustering by average linkage and visualized with TreeView version 1.6 (2002).

## Supporting Information

Figure S1 ChIP-Seq verification by classical ChIP. (**A)** Developmental changes of H3K4me2 occupancy for 10 example genes. (**B)** Developmental changes of H3K27me3 occupancy for 10 example genes.
(TIF)

Figure S2 Validation of antibodies used in ChIP-seq. Mouse retinal nuclear proteins (**A**) were separated by SDS-PAGE and each antibody gave only a single band of the expected mobility. The bottom panel (**B**) shows histones stained with Coomassie R250.
(TIF)

Table S1 Total Occupancies +/−2.5 Kb around TSS of genes in NCBI RefSeq.
(XLS)

Table S2 Genes without H3K4me3 and H3K27me3 accumulation on their TSS throughout retina maturation.
(XLS)

Table S3 Genome wide histone modification H3K4me2 and H3K27me3 in the TSS region of all olfactory receptor genes.
(XLS)

Table S4 Histone modification and gene expression profiles for genes up-regulated during retina development (rod dependent).
(XLS)

Table S5 Histone modification and gene expression profiles for genes down-regulated during retina development (cell proliferation enriched).
(XLS)

Table S6 DAVID GO analysis for genes down-regulated during later retina development.
(XLS)

Table S7 DAVID GO analysis for genes up-regulated during later retina development.
(XLS)

Table S8 Gene cluster with the same epigenetic signature as known rod-specific genes identified from whole genome and their expression in retina or eye as defined in the six listed data sources.
(XLS)

Table S9 DAVID GO analysis of the gene functions from Table S8.
(XLS)

Table S10 Genes expressed in retina cell types other than rod photoreceptors.
(XLS)

Table S11 Clustering H3K4me3 and H3K27me3 accumulation at TSS in the genes expressed in retina other than rod photoreceptors throughout retina maturation.
(XLS)

Table S12 Average histone occupancy of H3K4me2 and H3K27me3 in gene promoter region (−2.5 Kb) and whole gene body for up-regulated rod genes in Figure 7A.
(XLS)

Table S13 Average histone occupancy of H3K4me2 and H3K27me3 in gene promoter region (−2.5 Kb) and whole gene body for down-regulated genes in Figure 7B.
(XLS)

Table S14 Average histone occupancy of H3K4me2 and H3K27me3 in gene promoter region (−2.5 Kb) and whole gene body for non-rod retinal genes in Figure 7C.
(XLS)

Table S15 PCR Primers for ChIP-PCR confirmation.
(XLS)

Text S1 (PDF)
