# Supplementary figures and images for: Stage and Gene Specific Signatures Defined by Histones H3K4me2 and H3K27me3 Accompany Mammalian Retina Maturation In Vivo
Source: PLoS One. 2012 Oct 9;7(10):e46867. doi: 10.1371/journal.pone.0046867 (PMC3467275; doi:10.1371/journal.pone.0046867)

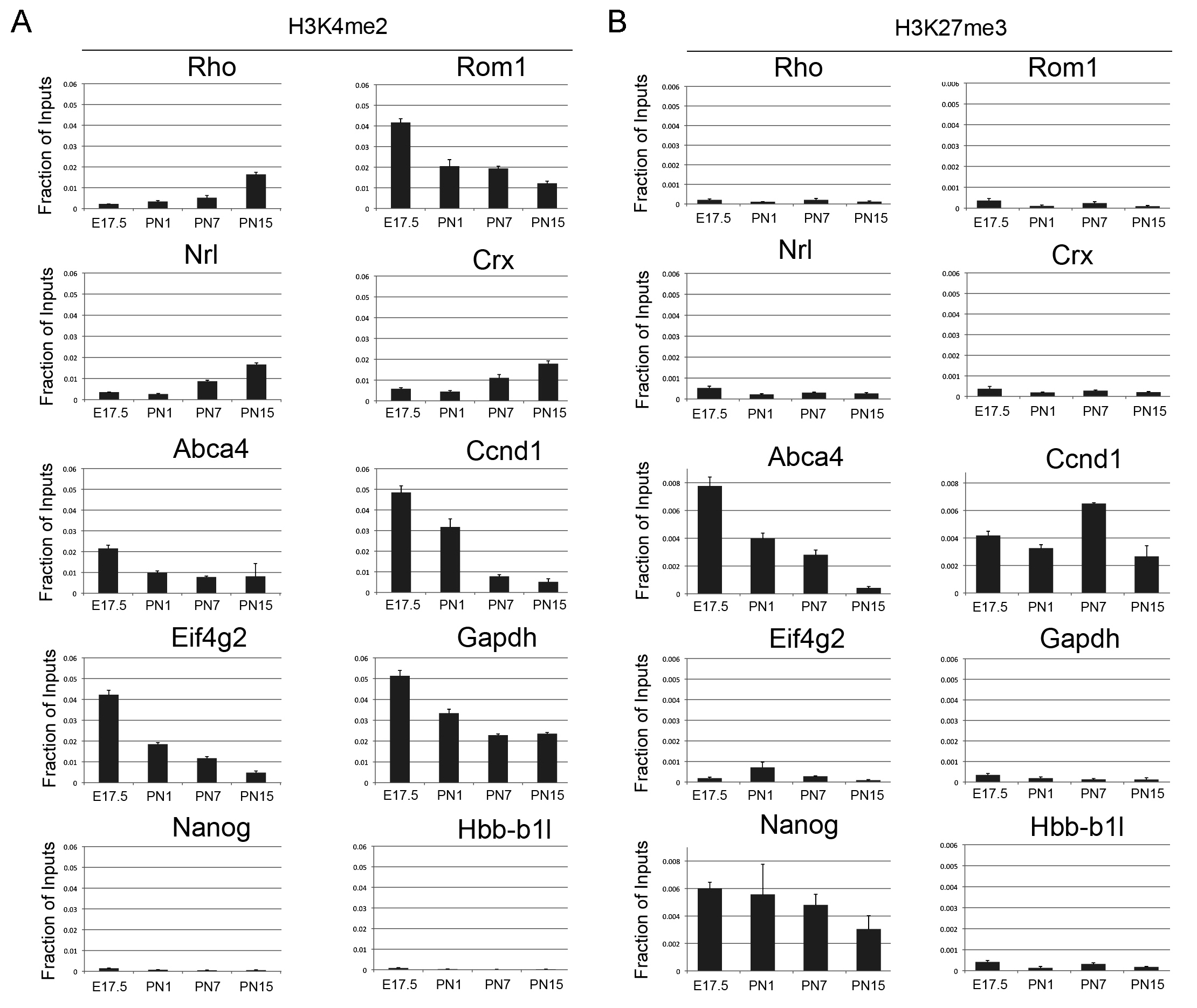

Supplement: Figure S1 — ChIP-Seq verification by classical ChIP. (A) Developmental changes of H3K4me2 occupancy for 10 example genes. (B) Developmental changes of H3K27me3 occupancy for 10 example genes. (TIF) [file pone.0046867.s001.tif]

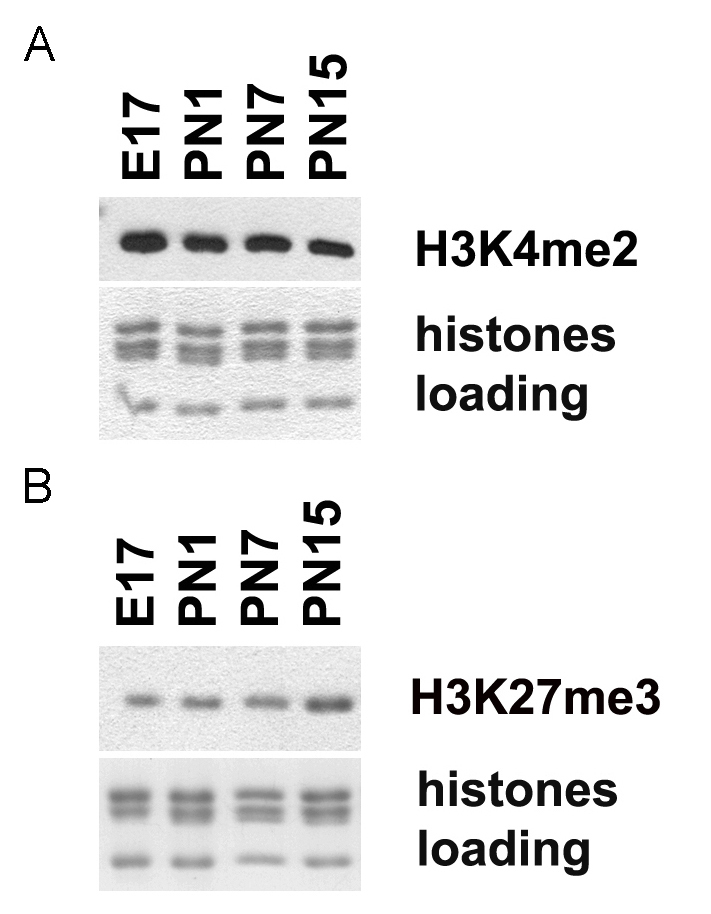

Supplement: Figure S2 — Validation of antibodies used in ChIP-seq. Mouse retinal nuclear proteins (A) were separated by SDS-PAGE and each antibody gave only a single band of the expected mobility. The bottom panel (B) shows histones stained with Coomassie R250. (TIF) [file pone.0046867.s002.tif]
