# Supplementary material for: Stage and Gene Specific Signatures Defined by Histones H3K4me2 and H3K27me3 Accompany Mammalian Retina Maturation In Vivo
Source: PLoS One. 2012 Oct 9;7(10):e46867. doi: 10.1371/journal.pone.0046867 (PMC3467275; doi:10.1371/journal.pone.0046867)
Supplement: Text S1 — (PDF) [file pone.0046867.s018.pdf]

Text S1

Materials and Methods

Mice. All animal experiments were conducted in accordance with NIH and ARVO guidelines and were approved by the Animal Care and Use Committee of Pennsylvania State University School of Medicine (Protocol # 2006-080 and 2009-061). C57BL/6j and *rd1/rd1*-Pde6b-RD1 mice[37] were purchased from the Jackson Laboratory (Bar Harbor, ME).

Antibodies and reagents. Chemicals were purchased from Fisher Scientific (Pittsburgh, PA), unless otherwise noted. Opsin[51] and SVP38[52] monoclonal antibodies have been described previously. Anti-H3K4me2 (07-030) and anti-H3K27me3 (07-449) were from Upstate (Charlottesville, VA) and were used before for ChIP[13,53] and passed validation (htt://compbio.med.harvard.edu/antibodies/)[54]. Anti-GFP antibodies were from Santa Cruz.

Immunohistochemistry. Methods were as previously described[55]. Antigen retrieval was achieved by boiling samples 5min in 10mM citrate buffer pH6.0. Sections were labeled with primary antibodies and secondary antibodies conjugated with FITC (Molecular Probes) or Texas Red (Jackson ImmunoResearch Inc.). Digital images were recorded using Olympus fluorescence or Olympus FV1000 confocal microscopes.

Nuclei isolation for ChIP-Seq and ChIP-qPCR. 20 mouse retinas were rapidly isolated and rinsed in PBS on ice. Cell suspensions in PBS were crosslinked with 1% formaldehyde for 15 min at room temperature, followed by quenching with 1M glycine, incubation on ice for 5min, and centrifugation for 7min at 4,000 rpm at 4˚C. For nuclei isolation the pellet was resuspended

in 1ml RSB buffer (10mMNaCl, 3mM $MgCl_2$, 19mM Hepes, pH7.5), with 1mM PMSF, 10ul protease inhibitors (PI) and 0.5% Igepal CA-630 (Sigma), incubated on ice for 20-30 min and centrifuged at 6,000 rpm for 7min at 4˚C.

For ChIP-Seq, nuclei were resuspended in 1ml RSB (PMSF+PI) and DNA concentrations measured spectrophotometrically. Micrococcal nuclease (MN) test digestions were carried out to determine the time interval needed to produce predominantly mononucleosomes and this was used for preparative digestion. For preparative micrococcal nuclease digestion, nuclei (0.5mg/ml DNA) was resuspended in 1 ml RSB, 0.5mM PMSF, 1mM $CaCl_2$, 2.5units/ml MN, incubated at 37˚C for 45-60 min and terminated by 5mM EDTA. Nuclei were centrifuged for 7 min at 7,500rpm, pellet was resuspended in 500ul L-CHIP buffer (1% SDS, 10mM EDTA, 50mM Tris-HCl pH8.0), 1mM PMSF and PI, sonicated twice at setting 3 for 10 sec on Sonic Dismembrator (Fisher Scientific, Model 100). The pellet of mononucleosomes was subjected to ChIP after pre-cleaning by centrifugation at 14,000rpm for 5 min. Protein concentration was adjusted to 1mg/ml with L-CHIP buffer. For ChIP-qPCR samples were treated similarly except that sonication to shear DNA to lengths of between 200 and 2,000bp was used.

Chromatin immunoprecipitation. Chromatin was diluted 10 fold in D-CHIP buffer and 5ug antibody was added and incubated with rotation overnight at 4˚C. Simultaneously 30ul protein A beads (Sigma) slurry were washed 2 times in washing buffer with 9:1 of D-CHIP (dilution buffer: 0.01% SDS, 1.1% Triton X-100, 1.2mM EDTA, 16.7mM Tris-HCl pH8.0, 167mM NaCl) and L-CHIP, resuspended in the same buffer with 500ug/ml salmon sperm DNA (Invitrogen) and 100ug/ml BSA (Invitrogen) and incubated on rotator overnight at 4˚C. Beads were washed 2 times with washing buffer, combined with the chromatin/antibody mix and rotated for 2 hours at

4˚C. Beads were washed 4 times with 1 ml LS-CHIP buffer (low salt buffer: 0.1% SDS, 1% Triton X-100, 2mM EDTA, 20mM Tris-HCl pH8.0, 150mM NaCl), 1 time with 1ml HS-CHIP buffer (high salt buffer: 0.1% SDS, 1% Triton X-100, 2mM EDTA, 20mM Tris-HCl pH8.0, 500mM NaCl) and eluted with 350ul of E-CHIP buffer (elution buffer: 1% SDS, 0.1M $NaHCO_3$) by rotating at room temp for 10min. Immunoprecipitate (IP) and 50ul of input chromatin (Input) were treated with 0.5mg/ml of Proteinase K (Roche) and RNaseA (Roche) at 37˚C for 30 min and uncrossliked at 65˚C overnight. DNA was extracted twice with phenol/chloroform, once with chloroform and ethanol precipitated with glycogen (Roche) and sodium acetate. DNA was dissolved in 50ul water and subjected to qPCR or was used to prepare libraries with IlluminaChIP-Seq DNA Sample Preparation Kit (IP-102-101). Quality and quantity of DNA in ChIP-Seq libraries were validated on Agilent Technologies 2100 Bioanalyzer. Libraries were sequenced on an Illumina Cluster Station and Genome Analyzer at Genomic Resource Center of The Rockefeller University.

qPCR. Quantitative real-time PCR was done according to Illumina Protocol Guide for qPCR Quantification (Illumina, Cat. # SY-930-1010) using primers listed in Supplemental Material (Supplementary Table 15). Triplicate samples were run on an iQ5 Multicolor Real Time PCR Detection System (Bio-Rad).

Data preparation. We used program Bowtie (version 0.12.4) to map all sequence reads to the mouse genome NCBI37/mm9. Sequence reads mapped to multiple genomic locations were excluded from subsequent analysis. The mid point of each unique-mapped reads was calculated with the assumption that all fragments are 190bp in size and were used for subsequent analysis.

One Bedfile was created for each sample by counting number of unique-mapped reads in each 100bp-window along all chromosomes normalized to the total unique-mapped reads in the sample. The final Bedfiles were uploaded to a website, and custom tracks were created in UCSC Genome Browser with links pointing to the real Bedfiles, that were used for specific gene mapping.

Tag distribution analysis in defined TSS region. To create the tag distribution around the TSS, we downloaded genomic data of RefSeq [35,56] (http://hgdownload.cse.ucsc.edu/goldenPath/mm9/database/refGene.txt), and counted the number of unique-mapped reads located in TSS +/-5Kb. Then the number of reads at each position was divided by the number of total reads in their regions. Smoothing function LOWESS in R package was used to calculate a smoothed density curve.

Detection of tag enriched region in genome. We applied a Poisson distribution-based model to detect enriched regions. We tested all windows in whole chromosome with pace of 100bp, windows with p-value below 1.0e-5 were collected as enriched, and overlapping windows were merged into single enriched region. We also tested the same enriched region on anti-GFP control samples and removed those regions from the enriched list if they were also significantly enriched in the GFP positive samples.

Detection of tag enriched location in individual genes. The reads from each experiment were mapped to mouse genome and analyzed with NextGENe software (version 2.10). Bed format files for specific genes were made for promoter area from +2.5Kb to TSS or for whole body of

the gene from TSS to TES and amount of reads were calculated for each genome interval at each developmental stage with NextGENe software. Normalized occupancy for each genome interval =(amount of reads x 5,000,000 reads x 1,000Kb)/(whole amount of reads in experiment x length of genome interval in Kb).

DAVID analysis. The Database for Annotation, Visualization and Integrated Discovery (DAVID[39,57]) v6.7 web-accessible programs (http://david.abcc.ncifcrf.gov) was used for GO functional analysis. Unigene ID of each gene from specific clusters is inputted into the web-based program each time to obtain gene-GO term enrichments score and p values under a default Searching Algorithm. The EASE Score and Benjamini values are used to evaluate gene functional enrichments.

Analysis of genes without H3K4me2 and H3K27me3 accumulation in retina. To have a better cutoff for the genes without H3K4me2 and H3K27me3 accumulation, a cluster of the normalized H3K4me2 and H3K27me3 dataset of the E17, PN1, PN7, and PN15 time-points were created using MatLab version R2011b based on Euclidean row distance. The average of the GFP controls (value of 9.12) was used as a background and normalized as an Euclidean distance of 8 dimensions (value of 25.80) to determine the cutoff of the Euclidean row distances indicating genes with no H3K4me2 or H3K27me3 hits.

Statistical analysis was done with Excel Data Analysis (Microsoft Excel 2007) and GraphPad Prizm 4 software. Cluster analysis[38] was done with Gene Cluster 3.0 software with hierarchical clustering by average linkage and visualized with TreeView version 1.6 (2002).
